# Bladder inflation to reduce hemorrhage secondary to a pelvic fracture

**DOI:** 10.1016/j.tcr.2024.101105

**Published:** 2024-09-14

**Authors:** Mark Fitzgerald, Tuan Phan, Sarah Fitzgerald, Yuewei Xiao, Madeline Green, Cecil Johnny, Joseph Mathew, Robert Gocentas, Warren Clements

**Affiliations:** a Trauma Service, The Alfred Hospital, Australia; b National Trauma Research Institute, Australia; c School of Translational Medicine, Monash University, Australia; d Interventional Radiology, The Alfred Hospital, Australia; e Emergency and Trauma Centre, The Alfred Hospital, Australia

**Keywords:** Pelvic fracture, Shock, Bladder inflation, Hemorrhage control

## Abstract

Bladder inflation may be a temporizing measure to tamponade pelvic bleeding in select trauma cases to bridge the patient to definitive interventions. Ultrasonographic confirmation of an intact bladder with an adjacent pelvic haematoma in a shocked adult with pelvic fracture is used for subject selection. An illustrative example of physiologic and interventional radiological control of pelvic bleeding following bladder inflation with sterile saline is presented.

Adult blunt multi-trauma patients with pelvic ring fractures are at risk of significant hemorrhage. Venous, arterial and medullary injuries with associated bleeding may be potentiated by an increased pelvic volume with ring disruption, as well as a reduced pressure effect from retroperitoneal and intra-pelvic organs on bleeding sites.

Various techniques are used to reduce intra-pelvic bleeding [1,2]. For shocked patients who have sustained major pelvic injuries with no other signs of urinary tract trauma and minimal urine in an intact bladder on initial FAST scan, we had previously advocated careful, aseptic Foley catheter insertion followed by bladder inflation with 500–600 ml of Normal Saline (NS) and subsequent catheter clamping to tamponade pelvic bleeding [3,4].

This case report illustrates the immediate physiologic effect and the CT imaging results of a case where this technique was utilized. Written permission from the case report was obtained from the patient using The Alfred Ethics and Research Committee Guidelines. No funding was required for, or supported this publication.

A 58-year-old male pedestrian arrived via EMS in the Trauma Center after being run over by a car whilst crossing the road. Bystanders reported a loss of consciousness. Attending paramedics had placed a pelvic binder as well as a cervical collar and had administered high flow oxygen and 100 ml IV of normal saline en route. On arrival the patient was alert and oriented, complaining of sternal, rib and pelvic pain, with a pulse rate of 105 per minute, blood pressure of 112/76 mmHg, respiratory rate of 16/min, an oxygen saturation of 93 % on high flow oxygen and a GCS of 15. Primary survey revealed decreased air entry into the left chest and reduced peripheral perfusion. Chest X-ray demonstrated bilateral rib fractures with subcutaneous emphysema. Pelvic X-ray demonstrated a type 1 left lateral compression fracture (Fig. 1). Ultrasound demonstrated no intraperitoneal or intrapericardial fluid, approximately 200 ml within the bladder and a left pelvic hematoma.

**Fig. 1:**
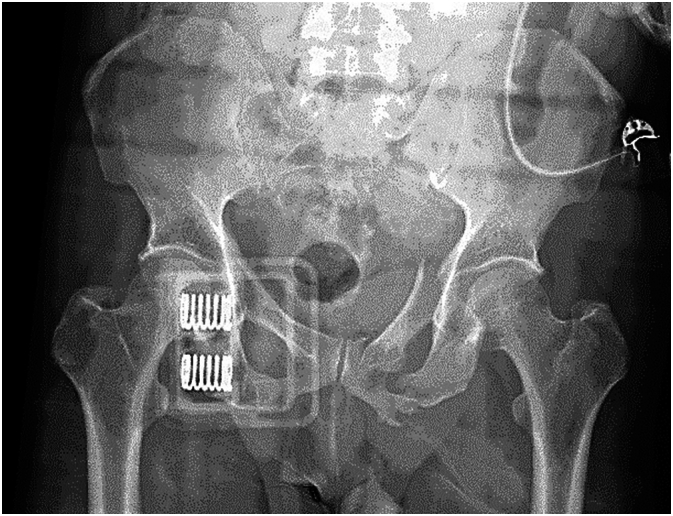
Pelvis X-ray during resuscitation, demonstrating left rami fractures and bladder displacement to the right. A pelvic binder is in situ.

A blood transfusion was commenced, and the patient was given Ketamine to facilitate bilateral chest drain insertion. A left radial arterial line was inserted. The patient was intubated to improve oxygenation and for safe CT scanning. The pelvic binder was undone, with subsequent X-ray demonstrating no signs of instability.

20 min after intubation and binder release, as the patent was about to be transferred for CT scanning, the systolic blood pressure dropped to 48 mmHg systolic, despite ongoing transfusion. The right sided pelvic hematoma had increased in size, without sonographic evidence of bladder rupture or intraperitoneal blood. The external genitalia appeared normal, without meatal blood.

The binder was reapplied. A 16 Fr Foley catheter was inserted into the bladder and 500 ml of sterile saline was aseptically inserted with a Toomey syringe under ultrasound control. The catheter was clamped. Shortly after the patient's blood pressure stabilized, with no evidence of ongoing bleeding.

CT scanning with the bladder inflated demonstrated a moderate volume left retroperitoneal hematoma, with displaced fractures of the left superior and inferior rami, and a displaced fracture of the left sacrum. There was no contrast extravasation on arterial or delayed phase (Fig. 2).

**Fig. 2:**
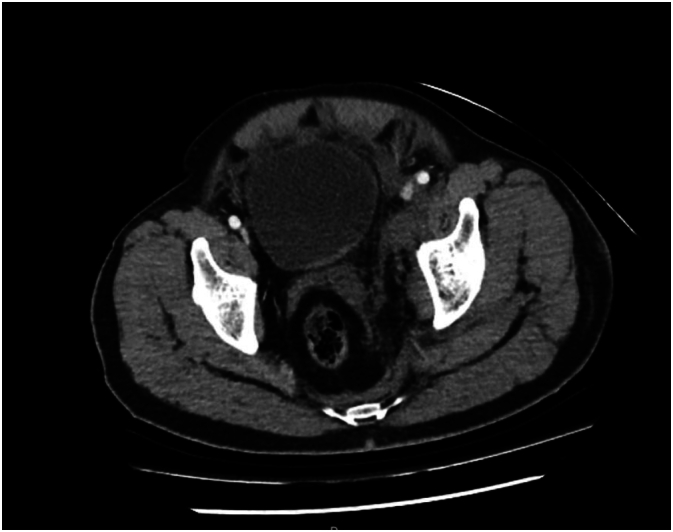
Axial CT Pelvis with inflated bladder and non-enhancing left hematoma.

The patient remained on the CT table, the catheter was unclamped, and the bladder drained. Once drained, repeat scanning demonstrated contrast extravasation adjacent to the fractured rami (Fig. 3). The patient subsequently become hypotensive, but stabilized once the bladder was reinflated. The patient was transferred to the interventional angiography suite and underwent Gelfoam® embolization of multiple distal branches of the anterior division of the internal iliac artery. Which controlled the bleeding. An IVC filter was inserted due to the presence of subarachnoid hemorrhage and the associated delay to anticoagulation. In the first 6 h following presentation, 11 units of red cell concentrate, 8 units of fresh frozen plasma, 10 units of cryoprecipitate and 2 units of pooled platelets were transfused. 4 units of red cell concentrate were further transfused in the 4 days post embolization.

**Fig. 3:**
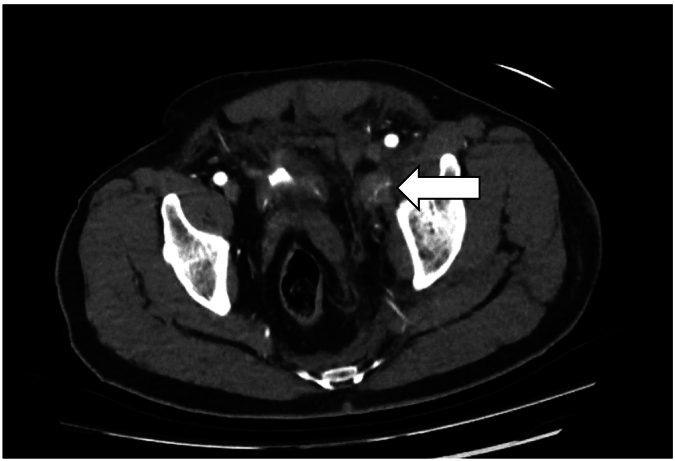
Axial CT Pelvis with deflated bladder and enhancing left hematoma (arrow).

The patients' injuries included a frontal subarachnoid hemorrhage, facial abrasions, an occipital condyle fracture, left C4 and right C6 transverse process fractures, multiple bilateral fractured ribs, a right pneumothorax, a left tension pneumothorax, a fractured left pelvis, a fractured left scapula and a fractured left humeral shaft.

Operative application of a left superior pubic ramus Matta pectineal plate (Stryker) and insertion of a left S1 Ilio-Sacral screw of the pelvis (Fig. 4) as well as fixation of the humerus was subsequently undertaken. The patient was well at discharge on day 15.

**Fig. 4:**
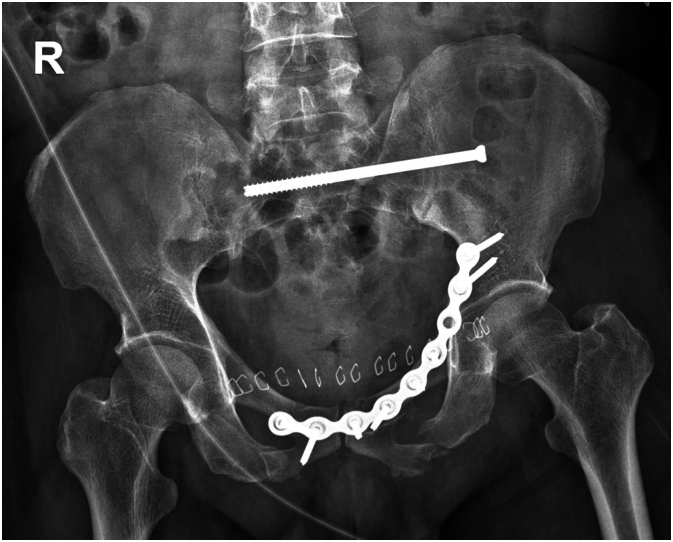
Post-operative X-ray.

Successful hemorrhage control from pelvic fractures requires a multimodal approach [1,2,5]. Bladder inflation in an anesthetized patient may be a useful adjunct in the initial resuscitation of unstable patients with pelvic fractures when used as temporizing measure to tamponade pelvic bleeding in select trauma cases - and to bridge the patient to definitive interventions, which in this case was Gelfoam® embolization.

The technique requires an intact urethra, and intact bladder (confirmed on ultrasound), ultrasound visualization of the bladder during inflation and suitable analgesia/anaesthesia.

This report is the first to graphically demonstrate successful tamponade of pelvic bleeding using bladder inflation.

## CRediT authorship contribution statement

**Mark Fitzgerald:** Writing – review & editing, Writing – original draft, Methodology, Investigation, Conceptualization. **Tuan Phan:** Writing – review & editing, Methodology. **Sarah Fitzgerald:** Writing – original draft, Visualization, Methodology, Data curation. **Yuewei Xiao:** Methodology. **Madeline Green:** Writing – review & editing, Formal analysis. **Cecil Johnny:** Writing – review & editing, Conceptualization. **Joseph Mathew:** Writing – review & editing, Methodology. **Robert Gocentas:** Writing – review & editing, Methodology. **Warren Clements:** Writing – review & editing.

## Declaration of competing interest

The declaration of Interests is within the letter to the Editor.

## References

[1] Fitzgerald M., Esser M., Russ M., Mathew J., Varma D., Wilkinson A. (2017). Pelvic trauma mortality reduced by integrated trauma care. Emerg. Med. Australas..

[2] Clements W., Dunne T., Clare S., Lukies M., Fitzgerald M., Mathew J. (2024). A retrospective observational study assessing mortality after pelvic trauma embolisation. J. Med. Imaging Radiat. Oncol..

[3] Huang S., Vohora A., Russ M.K., Mathew J.K., Johnny C.S., Stevens J., Fitzgerald M.C. (2015). Delaying urinary catheter insertion in the reception and resuscitation of blunt multitrauma and using a full bladder to tamponade pelvic bleeding. Injury.

[4] Lückhoff C., Mitra B., Cameron P.A., Fitzgerald M., Royce P. (2011). The diagnosis of acute urethral trauma. Injury.

[5] Harfouche M., Inaba K., Cannon J., Seamon M., Moore E., Scalea T., DuBose J. (2021). Patterns and outcomes of zone 3 REBOA use in the management of severe pelvic fractures: results from the AAST aortic occlusion for resuscitation in trauma and acute care surgery database. J. Trauma Acute Care Surg..

